# SUMO-2 Promotes mRNA Translation by Enhancing Interaction between eIF4E and eIF4G

**DOI:** 10.1371/journal.pone.0100457

**Published:** 2014-06-27

**Authors:** Li-zhao Chen, Xiang-yun Li, Hong Huang, Wei Xing, Wei Guo, Jing He, Zhi-ya Sun, An-xiong Luo, Hua-ping Liang, Jing Hu, Xiang Xu, Yun-sheng Xu, Zheng-guo Wang

**Affiliations:** 1 First department, State Key Laboratory of Trauma, Burn and Combined Injury, Research Institute of Surgery and Daping Hospital, Third Military Medical University, Chongqing, China; 2 Department of Neurosurgery, Research Institute of Surgery and Daping Hospital, Third Military Medical University, Chongqing, China; 3 Cell-based Biotherapy Center, Research Institute of Surgery and Daping Hospital, Third Military Medical University, Chongqing, China; 4 Fourth department, State Key Laboratory of Trauma, Burn and Combined Injury, Research Institute of Surgery and Daping Hospital, Third Military Medical University, Chongqing, China; 5 Department of Pharmacology and Chemical Biology, University of Pittsburgh Cancer Institute, University of Pittsburgh School of Medicine, Pittsburgh, Pennsylvania, United States of America; 6 Department of Dermatology, First Affiliated Hospital of Wenzhou Medical College, Wenzhou Zhejiang, China; Korea University, Korea, Republic of

## Abstract

Small ubiquitin-like modifier (SUMO) proteins regulate many important eukaryotic cellular processes through reversible covalent conjugation to target proteins. In addition to its many well-known biological consequences, like subcellular translocation of protein, subnuclear structure formation, and modulation of transcriptional activity, we show here that SUMO-2 also plays a role in mRNA translation. SUMO-2 promoted formation of the active eukaryotic initiation factor 4F (eIF4F) complex by enhancing interaction between Eukaryotic Initiation Factor 4E (eIF4E) and Eukaryotic Initiation Factor 4G (eIF4G), and induced translation of a subset of proteins, such as cyclinD1 and c-myc, which essential for cell proliferation and apoptosis. As expected, overexpression of SUMO-2 can partially cancel out the disrupting effect of 4EGI-1, a small molecule inhibitor of eIF4E/eIF4G interaction, on formation of the eIF4F complex, translation of the cap-dependent protein, cell proliferation and apoptosis. On the other hand, SUMO-2 knockdown via shRNA partially impaired cap-dependent translation and cell proliferation and promoted apoptosis. These results collectively suggest that SUMO-2 conjugation plays a crucial regulatory role in protein synthesis. Thus, this report might contribute to the basic understanding of mammalian protein translation and sheds some new light on the role of SUMO in this process.

## Introduction

Small ubiquitin-like modifiers (SUMO) are ubiquitin-related proteins that can be covalently conjugated to target proteins in cells to modify their function. To date, four SUMO isoforms encoded by separate genes, designated SUMO-1 to SUMO-4, have been identified in humans [1], [2]. The sequence identity and expression of these four SUMO molecules is highly variable. SUMO-2 and SUMO-3 are nearly identical, but share only 50% identity with SUMO-1 [3]–[5]. While SUMO-1, -2 and -3 are expressed ubiquitously, SUMO-4 seems to be expressed mainly in the kidney, lymph nodes and spleen.

Protein sumoylation is mediated by activating (E1), conjugating (E2) and ligating (E3) enzymes [6]. Ubc9 is the only identified SUMO E2 conjugating enzyme, which is sufficient for sumoylation. The E3 ligase promotes the efficiency of sumoylation and in some cases has been shown to direct SUMO conjugation onto non-consensus motifs [7]. Furthermore, sumoylation is reversible and is removed from targets by several specific SUMO proteases in an ATP-dependent manner [8].

SUMO modification has emerged as an important regulatory mechanism for protein activity, stability and localization. Most of the SUMO targets identified thus far are involved in various cellular processes, such as nucleocytoplasmic transport, transcriptional regulation, apoptosis, response to stress, and cell cycle progression [9]. Sumoylation regulates several aspects of gene expression, including DNA transcription, mRNA splicing and mRNA polyadenylation [7], [9], [10]. Furthermore, our recent study demonstrated that SUMO modification also regulates protein translation [11].

In eukaryotes, most proteins are synthesized through cap-dependent mRNA translation. A rate-limiting stepof this process is formation of the eIF4F complex containing eIF4E (cap-binding protein), eIF4A (ATP-dependent mRNA helicase) and eIF4G (scaffold protein) [12]. Binding of eIF4G to the cap structure of mRNA is competed by a small family of eIF4E-binding proteins (4E-BPs). 4E-BP1 is the most abundant member of the 4E-BP family. Its phosphorylation sites of Ser65 and Thr70 have been shown to participate in formation of the eIF4F complex. In particular, eIF4E phosphorylation at Ser209 and eIF4E SUMO conjugation by SUMO-1 seems to be important for initiation of cap-dependent translation [11], [13]. Furthermore, we found that overexpression of UBC9, the only identified SUMO E2 conjugating enzyme, dramatically increased expression of a cap-dependent luciferase reporter, but overexpression of SUMO-1 only slightly increased the expression of the luciferase reporter. Thus, we speculated that SUMO-2/3 isoform conjugations are involved in the regulation of cap-dependent mRNA translation. However, whether SUMO-2/3 conjugation plays a role in the regulation of cap-dependent mRNA translation and the innate mechanisms are still unclear.

In this study, we characterized the role of SUMO-2 conjugation in mRNA translation initiation through a SUMO-2 motif-negative mutation, overexpression and shRNA interference experiments, a translation reporter assay, and an inhibitor treatment. Furthermore, we studied the effect of regulation of mRNA translation by SUMO-2 on cell proliferation and apoptosis.

## Materials and Methods

### Cell culture and drug treatments

Human colorectal cancer HCT 116 cells were purchased from ATCC (ATCC, Manassas, VA, USA). Cells were grown in a humidified incubator with 5% CO2 at 37°C in McCoy's 5A Medium (Invitrogen) supplemented with 10% fetal bovine serum (FBS). For serum starvation and stimulation experiments, the cells were seeded and maintained in McCoy's 5A Medium plus 10% FBS. The following day, the cells were washed twice in Dulbecco's phosphate buffered saline (D-PBS) and maintained in McCoy's 5A Medium with 0.2% FBS. Twelve hours later, the cells were stimulated with or without 20% FBS for an additional 2 h. eIF4E/eIF4G Interaction Inhibitor (4EGI-1) (50 µM, Santa Cruz, CA) was added to the appropriate media at the indicated times for 24 h.

### Plasmids, Mutagenesis and Transfection

The PCR-amplified cDNAs encoding the processed forms of SUMO-1, SUMO-2, and SUMO-3 containing Gly-Gly at their C-termini were inserted into the pcDNA3-HA3 vector, which was described previously [11]. EcoR I (EcoR V for SUMO-1)/Apa I fragments were used to generate pcDNA3-HA3-SUMO-1/2/3 plasmids. The pcDNA3-HA3-SUMO-Δ1/2/3 plasmids lacking the C-terminal Gly-Gly were constructed using 3′-primers specifically designed with the corresponding mutation. The shRNA oligos were designed and subcloned into pLKO.1 (Puro) according to the Addgene Plasmid 10878 protocol. A negative control vector containing scrambled shRNA was constructed using the same method. Transient transfections were performed using PolyJet (SignaGen Laboratories, Ijamsville, MD) according to attached protocol. The cells were harvested 48 h after transfection. For the shRNA knockdown of SUMO-2, the cells were selected by puromycin for approximately 7 d. The gene-silencing effect was evaluated by Western blot analysis.

### Extract preparation, immunoprecipitation and Western blotting

The cells were washed twice with cold PBS and harvested in RIPA lysis buffer containing 1% NP-40, 50 mM Tris-base (pH 7.4), 2.5% sodium deoxycholate, 1 mM EDTA, 150 mM NaCl, and a protease inhibitor cocktail (Sigma) (1 pill/10 ml of lysis buffer). The cell debris were pelleted by centrifugation, and the protein concentration in the supernatant was measured using the Coomassie brilliant blue G-250 assay. For immunoprecipitation to detect the interactions between eIF4E, eIF4G and eIF4A, a total of 300 µg of protein was then mixed with the corresponding antibody and rotated overnight at 4°C. The immunocomplexes were recovered by incubating with Protein G-Sepharose for 4 h, and the resin was washed five times with RIPA lysis buffer. The samples were separated using sodium dodecyl sulfate-polyacrylamide gel electrophoresis (SDS-PAGE) and transferred to polyvinylidene difluoride membranes. Immunoblotting was performed with antibodies against Mammalian Target of Rapamycin (mTOR), phospho-Ser2448 mTOR, p70 S6 kinase, phospho-Thr421/Ser424 p70 S6 kinase, 4E-BP1, phospho-Thr37/46 4E-BP1, phospho-Thr70 4E-BP1, S6, phospho-Ser235/236 S6, eIF4E, and phospho-Ser209 eIF4E (Cell Signaling, Beverley, MA); eIF4AI/II, HA, c-myc (Santa Cruz, Inc., CA); eIF4G1/eIF4GI (Bethyl Laboratories, Inc.); and beta-actin (Abcam, Inc., Cambridge, MA).

### Cap-dependent reporter gene assay

As previously described [14], transcription from reporter gene pYIC DNA produces a bicistronic mRNA encoding epitope-tagged yellow fluorescent protein (EYFP) and cyan fluorescent protein. EYFP translation depends on 5′ cap sequence,so if a protein interfere with the cap-dependent pathway, EYFP translation is reduced. As EYFP were tagged with three c-myc epitope tags, we can use the corresponding antibody against c-myc or fluorescence intensity to indicate the expression of EYFP, and the results will help us to decide whether the stimulus interferes with protein translation through cap -dependent pathway.

### m7GTP Pull-down Assay

The method has been described previously [15]. Briefly, 10 µl of m7GTP Sepharose beads (Amersham) was washed with 500 µl of PBS three times, added to 300 µg of total protein from the cell lysates pre-cleared with Sepharose resin without m7GTP for 1 hour, and rotated overnight at 4°C. The beads were washed four to five times with PBS. The samples were then denatured, and the supernatants were loaded into SDS-PAGE for Western blot analysis.

### Reverse Transcription and PCR (RT-PCR)

Total RNA was isolated using the TRIzol reagent (Invitrogen, Cincinnati, OH, USA) according to the manufacturer's protocol. RNA (1 µg) was reverse transcribed using Superscript II reverse transcriptase with an oligo (dT) primer (Invitrogen, Carlsbad, CA). The primers used to amplify the 281 bases of the human LUC gene were 5′-CCGGGAAAACGCTGGGCGTTA-3′ and 5′-ACCTGCGACACCTGCGTCGA-3′. The 202 bases of the housekeeping gene β-actin were amplified using the following primers: 5′-CACCCGCCGCCAGCTCAC-3′ and 5′-CTTGCTCTGGGCCTCGTCGC-3′.

### Luciferase Activity Assay

The cells were seeded in 24-well plates and co-transfected with the pcDNA3-HA3 reporter plasmid and the target plasmid using the transfection reagent (5∶1 ratio; Roche Applied Science) following the manufacturer's protocol. Twenty-four hours later, the cells were lysed and subjected to a luciferase activity assay using a luciferase assay system (Promega) in a luminometer. The relative luciferase activity was normalized to β-galactosidase activity, which was measured as described previously [16].

### Cell growth rate analysis

In these experiments, 2×10^4^ cells were plated in each well of 48-well plates and cultured for a 48 h period. Cell viability was determined using the MTT assay [17]. Breifly, 50 µl of 5 mg/ml MTT in PBS was added to each well and the plates incubated at 37°C for a further 4 h. The media as then removed and the purple formazan crystals were dissolved in 150 µl dimethyl sulfoxide. After the plate was agitated on a plate mixer, the optical densities were read at 490 nm with a Bio-RAD microplate reader.

### Apoptosis Assay

Serum starvation or 4EGI-1 induced apoptosis was assessed by flow cytometric analysis of quantitate Annexin V-positive and propidium iodide-positive cells. The HCT116 cells were transfected with the scrambled shRNA or SUMO-2 shRNA. The stable cell lines were established by puromycin (1 ng/µl) selection. The stable cell lines were incubated in McCoy's 5A Medium containing 0.1% FBS for 72 h. After serum starvation, the attached and floating cells were harvested. The cells were first stained with propidium iodide and then with Annexin V-Alexa Fluor 488 conjugate (Invitrogen) according to the manufacturer's instructions. Flow cytometry was used to quantify the Annexin V-positive and propidium iodide-positive cells. A minimum of 30,000 cells per measurement from triplicate samples were analyzed for each experiment.

### Statistical Analysis

The band intensity for RT-PCR assay and western-blot assay was analyzed by using the UN-SCAN-IT gel-graph digitizing software. Results are expressed as means ± SE. Statistical analyses were performed by one-way analysis of variance followed by Dunnett's post hoc or Tukey's multiple comparison test using the data obtained from three or five independent experiments.

## Results

### SUMO-2 promotes cap-dependent protein translation

**Figure 1:**
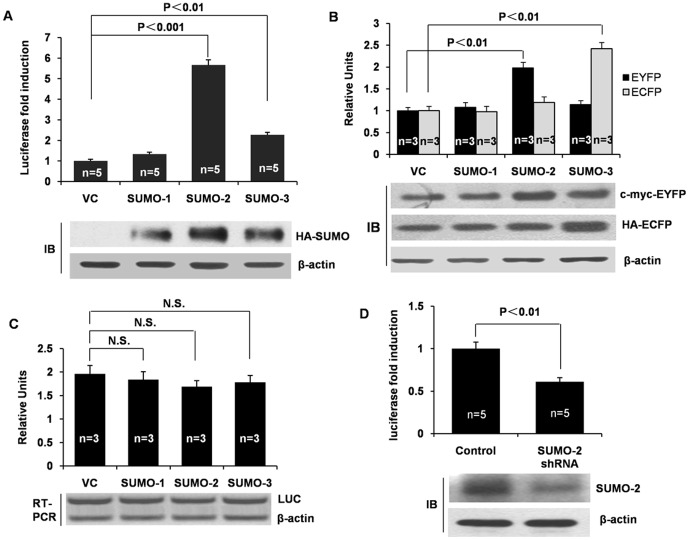
SUMO-2 Promotes cap-dependent mRNA translation. (A) The effects of the overexpressed SUMO isoforms on cap-dependent LUC activities. HCT116 cells were co-transfected with individual SUMO isoforms along with a luciferase reporter gene. Twenty-four hours later, the cells were lysed, and the luciferase activity was measured using a luciferase assay kit (Promega). The absolute relative light units values were in the 10^7^–10^8^ range. The expression levels of SUMO proteins in the cell lysates are also shown. (B) SUMO-2 activates cap-dependent EYFP expression. HCT116 cells were co-transfected with pYIC and SUMO-2 or SUMO-3. EYFP and ECFP were detected using c-myc and HA-tag antibodies, respectively. (C) The effect of the overexpression of the SUMO isoforms on LUC mRNA transcription in HCT116 cells. Human HCT116 cells were transfected with each of the SUMO isoforms. Twenty four hours after transfection, the cells were harvested, and the whole cell lysates were used for the RT-PCR assays. The reporter gene LUC was detected. β-actin protein was used as the loading control. (D) The shRNA knockdown of SUMO-2 reduces LUC expression. HCT116 stable cells lines with an empty vector or SUMO2 shRNA were selected on the basis of puromycin resistance, then transiently transfected with the luciferase reporter gene. Twenty-four hours after transfection, the cells were harvested for the luciferase assay. The data is an average of 5 measurements of a single clone. The knockdown effects are also shown. The error bars represent the S.E. LUC, luciferase; shRNA, short hairpin RNA; SUMO, small ubiquitin-related modifier; RT-PCR, reverse transcription and polymerase chain reaction; Vc, vector control.

To date, little attention has been given to the impact of the individual SUMO paralogues, SUMO-1, SUMO-2 and SUMO-3, on protein translation. To examine whether SUMOs affects protein translation, we surveyed cap-dependent luciferase activity, cap-dependent EYFP expression and IRES-dependent ECFP expression under normal and SUMO over-expression conditions. As shown in Fig. 1A, overexpression of SUMO-2 and SUMO-3 by transient transfection induced a striking increase in cap-dependent luciferase activity (almost 5-fold for SUMO-2 and 2.5-fold for SUMO-3), while SUMO-1 had little effect on cap-dependent translation (Fig. 1A). Furthermore, overexpression of SUMO-2 also significantly promoted expression of cap-dependent EYFP, but not affected the expression of IRES-dependent ECFP. On the other hand, the overexpression of SUMO-3 markedly increased expression of IRES-dependent ECFP, but did not affect cap-dependent EYFP (Fig. 1B).

To evaluate whether SUMO-2 promotes protein translation by increasing total mRNA level, we ran RT-PCR for LUC genes. However, there was no significant change between the control and experimental groups (Fig. 1C). SUMO-2 also didn't change mRNA level of ECFP and EYFP (data no shown). In contrast with the effect of overexpression, SUMO-2 knockdown via SUMO-2 shRNA reduced cap-dependent luciferase activity (Fig. 1D). Collectively, these data indicate that sumoylation by SUMO-2 plays a positive role in cap-dependent protein translation.

### SUMO-2 conjugation is a prerequisite for activating cap-dependent protein translation

**Figure 2:**
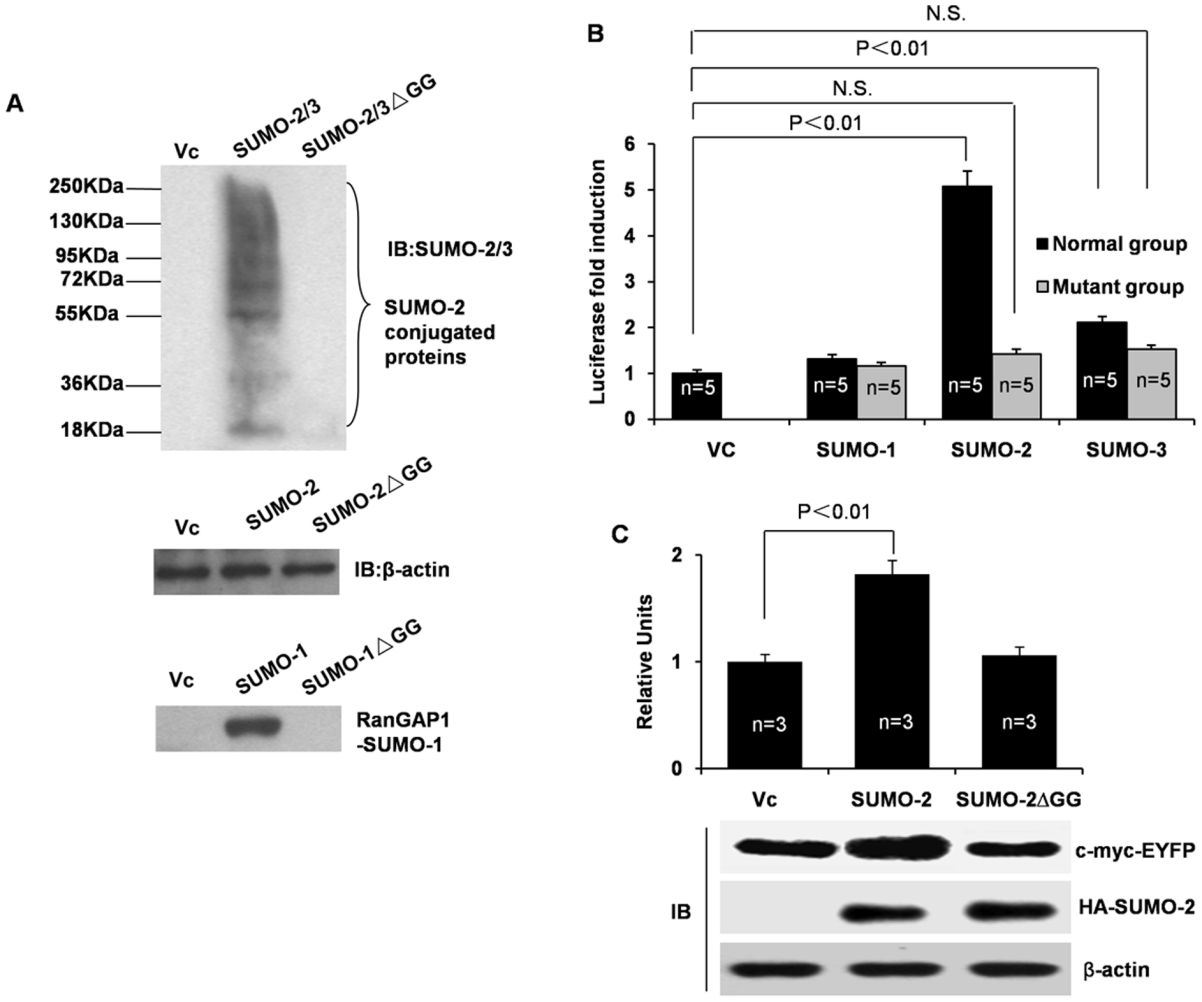
The effects of SUMO-2 on cap-dependent protein translation are dependent on SUMO-2 conjugation. (A) The SUMO mutants are unable to be covalently conjugated. HCT116 cells were transfected with the SUMO-2 isoforms and SUMO-2ΔGGmutant, respectively. The expression levels of conjugated SUMO-2 proteins in the cell lysates are detected byanti-SUMO-2/3 antibody (the 97% high identity between SUMO-2 and SUMO-3). Positive control lower panels, SUMO-1 conjugating RanGAP1 are detected by anti-SUMO-1 antibody. (B) The non-conjugatable form of SUMO-2 had no effect on cap-dependent LUC translation. HCT116 cells were co-transfected with a luciferase reporter gene and each of the SUMO isoforms or the corresponding mutant. Twenty-four hours later, the cells were harvested for a luciferase assay. The absolute luciferase relative light values were in the 10^7^–10^8^ range. Statistics were performed using a one-way analysis of variance followed by Tukey's multiple comparison test using the data obtained from five independent experiments. (C) The effects of the non-conjugatable SUMO-2 mutant on cap-dependent EYFP protein expression. The empty vector, SUMO-2, and SUMO-2ΔGG were co-transfected with pYIC in HCT116 cells. EYFP was detected using anti-c-myc antibody. The expression level of SUMO-2 and its mutant protein are also shown. SUMO-2ΔGG, the non-conjugatable form of SUMO-2that lacks di-glycine.

To exclude the possibility that SUMO-2 exerts its effect through non-covalent modifications, we made SUMO non-conjugatable mutants (mutants lacking the C-terminal Gly-Gly through which SUMOs congjugate with the target protein) and used these mutants to differentiate whether it was through covalent or non-covalent conjugation that SUMO promoted mRNA translation. In HCT116 cells, RanGAP1 is a major substrate of SUMO-1 and SUMO-1 antibody recognizes covalently conjugation RanGAP1-SUMO-1. Since SUMO-2 or SUMO-3 themselves can be sumoylated because they contain an internal SUMO consensus modification site, poly-SUMO-2 or SUMO-3 chains on SUMO-modified substrates can be generated. Fig. 2A confirmed that the SUMO mutants are unable to be covalently conjugated.The cap-dependent reporterassay using the luciferase reporter (Fig. 2B) and the EYFP reporter (Fig. 2C) both showed that SUMO-2/3 non-conjugatable mutants lost the ability to induce cap-dependent mRNA translation. These results suggested that cap-dependent mRNA translation was induced mainly by covalent SUMO conjugation to certain target proteins.

### SUMO-2 promotes formation of the eIF4F complex by enhancing interaction between eIF4E and eIF4G

**Figure 3:**
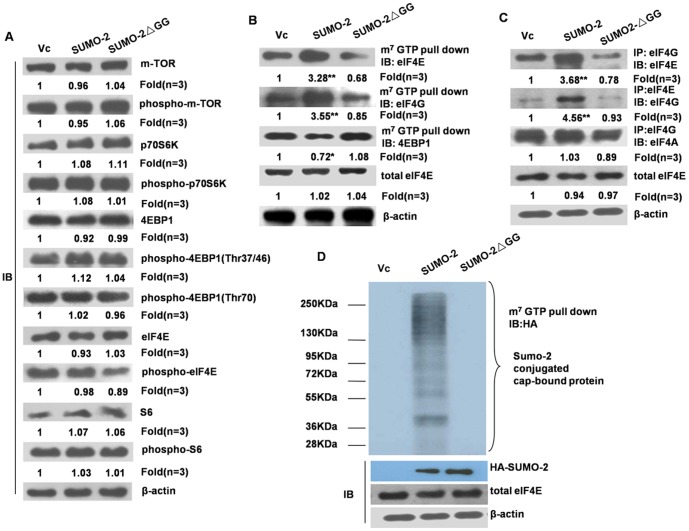
The effect of SUMO-2 on protein translation is achieved through enhancing the interaction between eIF4E and eIF4G. (A) The overexpression of SUMO-2 didn't activate the mTORC1 pathway. The empty vector, HA-SUMO-2, or HA-SUMO-2ΔGG were transfected into HCT116 cells. The cell lysates were used for immunoblot analysis of the total and phosphorylated level of key molecules in the mTOR pathway. (B) SUMO-2 promotes the formation of the eIF4F complex. The HCT116 cells were transfected with the empty vector, wild-type SUMO-2 or SUMO-2ΔGG. Twenty-four hours after transfection, the cells were harvested and lysed for the m7GTP pull-down assays. The cap-bound eIF4E, eIF4G, and 4EBP1 were eluted from the 7-methyl-GTP (m7GTP)-Sepharose resin using 7-methyl-GDP (m7GDP) and detected by immunoblotting with the corresponding antibodies. (C) SUMO-2 promotes the interaction between eIF4E and eIF4G. The HCT116 cells were transfected with the empty vector, wild-type SUMO-2 or SUMO-2ΔGG. Twenty-four hours after transfection, the cells were harvested and lysed for the co-immunoprecipitation assays. The immunoprecipitated eIF4E or eIF4A1/2- was blotted with eIF4G, and the immunoprecipitated eIF4G was blotted with eIF4E. (D) SUMO-2 is directly conjugated to the eIF4F complex. Processed the same way as (B), but immunoblotted with HA antibody. eIF, eukaryotic translation initiation factor. *, *p*<0.05 *vs* control; **, *p*<0.01 *vs* control.

Next, we investigated the mechanisms underlying the SUMO-2 induced cap-dependent mRNA translation. Firstly, we analyzed whether SUMO-2 promotes protein translation through mTORC1 signaling by over-expressing HA-SUMO-2 and non-conjugatable HA-SUMO-2ΔGG in HCT116 cells. And it was found that the whole mTOR pathway, including the total expression and phosphorylation of key molecules (such as m-TOR, p70S6K, S6, 4EBP1 and eIF4E) in the mTORC1 pathway, showed no obvious changes after overexpression of SUMO-2 or its non-conjugatable form (Fig. 3A).

Then, we examined the effect of SUMO-2 on interaction between eIF4E, eIF4A and eIF4G. As expected, the amount of eIF4E and eIF4G in the eIF4F complex increased markedly following SUMO-2 overexpression while 4EBP-1 significantly decreased (Fig. 3B). Furthermore, interactions between eIF4E and eIF4G were also enhanced, while the interaction between eIF4G and eIF4A was not affected (Fig. 3C).

In order to further study whether SUMO-2 promotes formation of eIF4F complex directly, we preformed m7GTP pull-down assay and immunoblotted with HA antibody. Intriguingly, when we overexpressed SUMO-2, bands from about 36 kDa to the top were detected, while the other two groups didn't show the same phenomenon (Figure. 3D). We first considered if the three major components of eIF4F complex can be sumoylated by SUMO-2. We know that bands below 130 kDa were not eIF4G-SUMO-2 for the molecular weight of eIF4G is 220 kDa. However, we can't exclude eIF4G may be sumoylated. Then we demonstrated if eIF4E and eIF4A can be modified by SUMO-2 by immunoprecipitation, but the results turned out to be negative (data not shown). For eIF4F complex contains dozens of other components, it is possible that the substrates of SUMO-2 existing in eIF4F that were not examined through above experiments, so it suggested that more experiment need to be done to find substrate protein of SUMO-2 in eIF4F complex.

### SUMO-2 rescues the inhibiting effects of 4EGI-1 on the interaction between eIF4E and eIF4G

**Figure 4:**
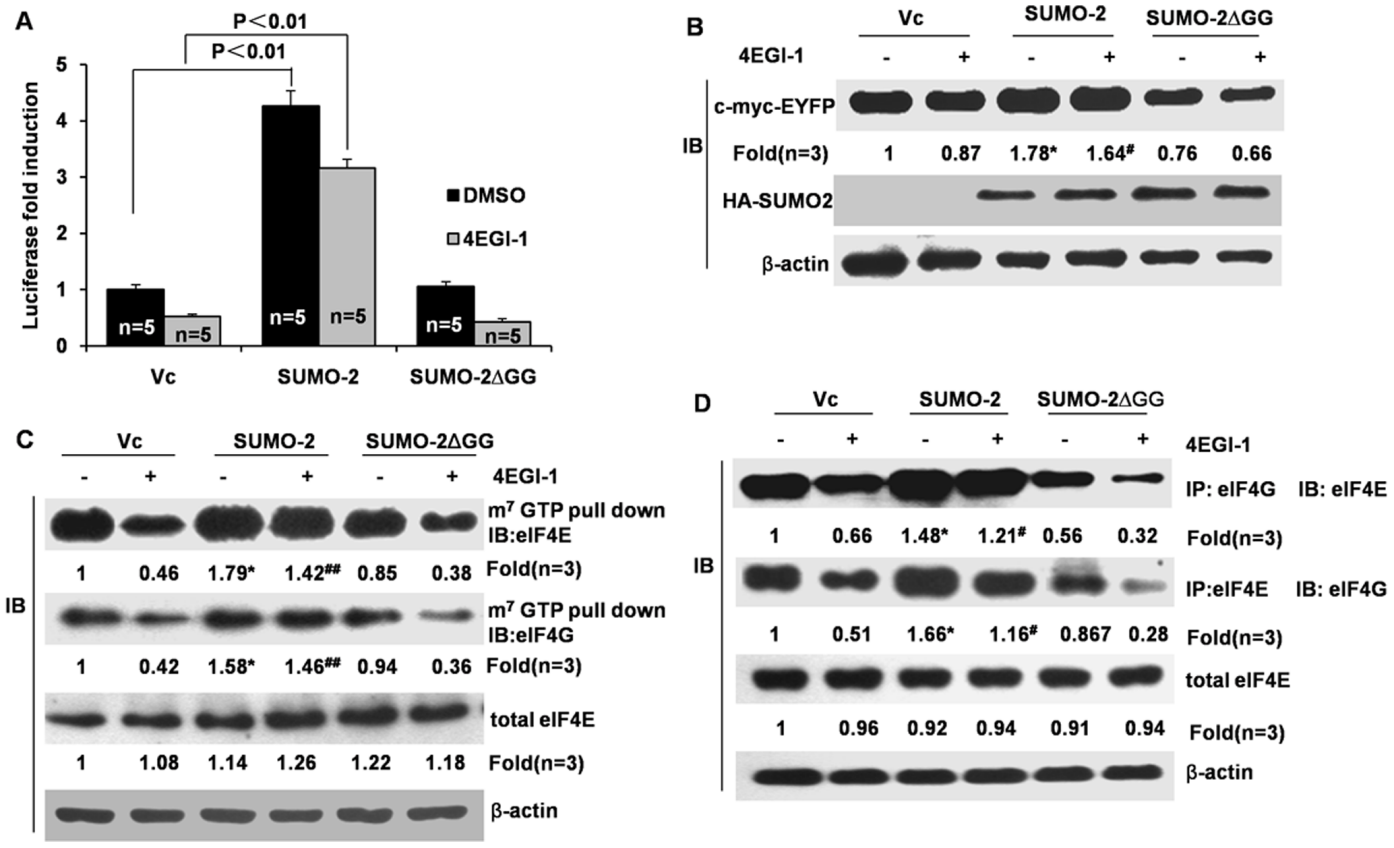
SUMO-2 partially rescues the effect of 4EGI-1 on cap-dependent luciferase translation and the interaction between eIF4E and eIF4G. HCT116 cells were co-transfected with LUC (A) or pYIC (B) with Vc, wild-type SUMO-2 or SUMO-2ΔGG. The cells were treated with 4EGI-1 (50 µM) for 24 h immediately after transient transfection. (A) The cells were harvested for the luciferase assay. (B) The cell lysates were detected for EYFP expression. HCT116 cells were transfected with Vc, wild-type SUMO-2 or SUMO-2ΔGG. Twenty-four hours post-transfection, the cells were treated with 4EGI-1 (50 µM) for another 24 h. (C) The pull-down assays were conducted. (D) The interaction between eIF4E and eIF4G was evaluated using a co-immunoprecipitation method. *, *p*<0.05 *vs* 4EGI-1-untreated control; **, *p*<0.01 *vs* 4EGI-1-untreated control; ^#^, *p*<0.05 *vs* 4EGI-1-untreated control; ^##^, *p*<0.01 *vs* 4EGI-1-untreated control.

To further confirm the idea that SUMO-2 promotes mRNA translation by enhancing the interaction between eIF4E and eIF4G, we used 4EGI-1, an eIF4E/eIF4G interaction inhibitor, to interfere with the interaction between eIF4G and eIF4E. Using m7GTP pull-down and an immunoprecipitation assay, we found that 4EGI-1 indeed disrupted the formation of the eIF4F complex by inhibiting the interaction between eIF4G and eIF4E, as previously reported [18] (Fig. 4A and B). Intriguingly, overexpression of SUMO-2 partially inhibited the effect of 4EGI-1 on interaction between eIF4E and eIF4G and increased formation of eIF4F complex, while SUMO-2ΔGG had no such effect (Fig. 4A and B). Furthermore, 4EGI-1 also inhibited cap-dependent LUC and EYFP expression, and SUMO-2 overexpression partially reduced the inhibitory effect of 4EGI-1 on LUC and EYFP expression (Fig. 4C and D). Taken together, our data indicate that overexpression of SUMO-2 partially induces cap-dependent mRNA translation by promoting the interaction between eIF4E and eIF4G and enhancing formation of active eIF4F complexes.

### SUMO-2 upregulates expression of two cap-dependent target proteins cyclin D1 and c-myc

**Figure 5:**
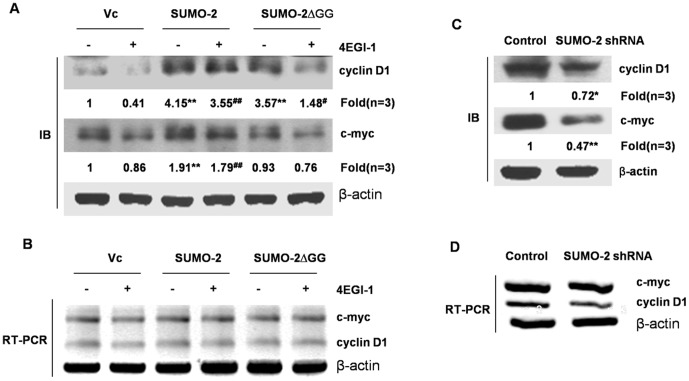
The effects of SUMO-2 on cap-dependent target proteins cyclin D1 and c-myc translation. HCT116 cells were transiently transfected with empty vector, wild-type SUMO-2 or SUMO-2ΔGG and cultured for 24 h. Then, the cells were treated with 4EGI-1 (50 µM) for another 24 h and lysed for immunoblotting and RT-PCR. The control group was treated with DMSO. (A) The effect of the overexpression of SUMO-2 on the expression of cyclin D1 and c-myc protein. **, *p*<0.01 *vs* 4EGI-1-untreated control; ^#^, *p*<0.05 *vs* 4EGI-1-untreated control; ^##^, *p*<0.01 *vs* 4EGI-1-untreated control. (B) The effect of the overexpression of SUMO-2 on cytosolic cyclin D1 and c-myc mRNAs levels. (C) The effect of SUMO-2 knockdown on the translation of the cap-dependent target proteins cyclin D1 and c-myc. *, *p*<0.05 *vs* control; **, *p*<0.01 *vs* control. (D) The effect of SUMO-2 knockdown on cytosolic cyclin D1 and c-myc mRNAs levels. Stable SUMO-2 knockdown cell lines were established by puromycin (1 ng/µl) selection. The cells were cultured in the puromycin free mediums for 24 h. The cells were then lysed and immunoblot and RT-PCR assays were performed using the same methods as in A and B.

Given that SUMO-2 substantially increases interaction between eIF4E and eIF4G, we hypothesized that expression of cap-dependent mRNA will also be promoted. To confirm this hypothesis, we chose two typical cap-dependent proteins, cyclin D1 and c-myc. We first determined the effect of 4EGI-1 on expression of c-myc and cyclin D1. Consistent with our hypothesis, 4EGI-1 at a concentration of 50 µM inhibits the expression of cap-dependent proteins, and overexpression of SUMO-2 can partially reverse this inhibitory effect (Fig. 5A). Furthermore, neither SUMO-2 nor 4EGI-1 affected the cytoplasmic mRNA levels of the target genes (Fig. 5B), thus excluding the possibility that SUMO-2 or 4EGI-1 alters gene transcription or mRNA transport of the genes. On the contrary, expression of c-myc and cyclin D1 were significantly inhibited when SUMO-2 was reduced by SUMO-2 shRNA (Fig. 5C), while their transcriptional levels were also not affected (Fig. 5D). These observations suggest that SUMO-2 upregulates two cap-dependent proteins c-myc and cyclin D1 translation.

### SUMO-2 promotes cell proliferation and inhibits apoptosis

**Figure 6:**
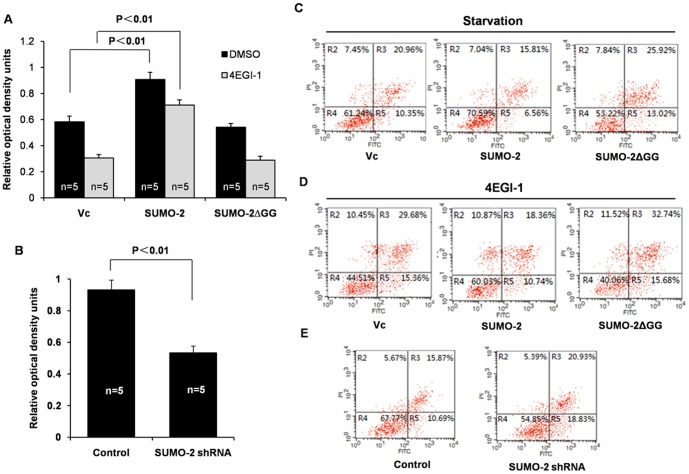
The effect of SUMO-2 on cell proliferation and apoptosis. (A) Overexpression of SUMO-2 promotes cell proliferation. The HCT-116 cells were infected with empty vector, wild-type SUMO-2, or SUMO-2ΔGG mutant. Twenty-four hours after transfection, the cells were treated with or without 50 µM 4EGI-1 for another 24 h. Then, cell proliferation was evaluated using an MTT assay. (B) shRNA knockdown of SUMO-2 inhibits cell proliferation. Stable SUMO-2 knockdown cell lines were established by puromycin (1 ng/µl) selection. The cells were cultured in the puromycin free medium for 24 h, then evaluated with MTT assay. (C) Overexpression of SUMO-2 inhibits the apoptosis induced by starvation. Following the same treatment described for *A*, the cells were then starved for 72 h, and apoptosis was measured using Annexin V staining. (D) Overexpression of SUMO-2 inhibits apoptosis induced by 4EGI-1. After the same treatment described for *A*, cell apoptosis as measured by using Annexin V staining. (E) The effect of SUMO-2 knockdown on apoptosis. The percent in the upper left represents the dead cells, while the percent in the upper right represents the non-viable apoptotic cells and dead cells. The percent in the lower right represents the viable apoptotic cells.

During culture of SUMO-2 silenced cells, we noticed that cells in the silenced group have much lower rates of cell growth, which was consistent witha previous study [19]. Thus, we assessed cell proliferation and apoptosis. As expected, overexpression of SUMO-2, but not SUMO-2ΔGG, significantly promotes cell proliferation and partially cancels out the inhibitory effect of 4EGI-1 on HCT116 cell proliferation (Fig. 6A). On the other hand, growth of SUMO-2-silenced HCT116 cells was markedly inhibited (Fig. 6B). Furthermore, transient overexpression of SUMO-2 markedly reduced HCT116 cell apoptosis induced by 4EGI-1 or starvation (Fig. 6, C and D). However, SUMO-2-silencing HCT116 cells showed higher rates of apoptosis (Fig. 6E). Taken together, these data indicate that SUMO-2 promotes cell proliferation and inhibits apoptosis.

## Discussion

Considering the remarkably extensive influence of sumoylation in cells, it is important to identify whether the components in sumoylation circulation impact protein translation. As previously mentioned, both SUMO-1 and Ubc9 can promote protein translation, and we suspected that SUMO-2 or SUMO-3 may also play a part in mRNA translation. In this study, we provided evidences to show that SUMO-2 can promote cap-dependent protein translation, while SUMO-3 seems to be involved in IRES-dependent protein translation. Despite that the high homology of the two isoforms brings great difficulty to characterization of the functional specificity of the two proteins, our study showed that SUMO-2 and SUMO-3 may play different roles in protein translation. The underlying mechanisms need further investigation.

So what is the molecular basis of the SUMO-2 induced activation of mRNA translation? We have four speculations. Firstly, proteomic analyses have revealed that many of the SUMO conjugation target proteins are transcription factors or other nuclear proteins that modulate gene expression [20]. We also know that mRNA levels of many known genes were significantly up- or down-regulated in SUMO-2/-3 miRNA overexpressing cells [19]. Thus, we examined whether SUMOs have effects on total mRNA. However, total mRNA proved to be unchanged. Secondly, the mTORC1 pathway is important for regulation of mRNA translation and integrates various signals, such as nutrients, growth factors, energy, and stress, to regulate cell growth and proliferation. However, overexpression of SUMO-2 has little effect on the mTORC1 pathway. Thirdly, cap-dependent translation is a fundamental operation in almost all aspects of cell functions, and one of the rate-limiting step in cap-dependent translation is the formation of the eIF4F complex containing eIF4E (cap-binding protein), eIF4A (ATP-dependent mRNA helicase) and eIF4G (scaffold protein). We found that the underlying mechanism for this result is the significantly enhanced interaction between eIF4E and eIF4G. The eIF4E/eIF4G interaction inhibitor 4EGI-1 can effectively inhibit the formation of active eIF4F complexes. After we treated HCT116 cells with 4EGI-1, the interaction between eIF4E and eIF4G was reduced and this effect was partly impaired when SUMO-2 was overexpressed. However, the effect was not reduced to the same level as the control groups. This may be because 4EGI-1 [21] and SUMO-2 [22] both have complicated networks within cells. Fourthly, we found that SUMO-2 promotes mRNA translation through covalent conjugation. Although the exact component of eIF4F complex modified by SUMO-2 is yet to be identified despite exclusion of three major components, our results did indicate that SUMO-2 modification is involved in the formation of eIF4F complex and promotes mRNA translation through the translational machinery.

shRNA was also used in this study to elucidate the effects of silencing of SUMO expression on global protein expression. As expected, after shRNA knockdown of SUMO-2, cap-dependent LUC and EYFP protein translation decreased, which again confirmed a role of SUMO-2 in protein translation. The shRNA knockdown of SUMO-2 partially inhibited protein translation and showed that SUMO-2 promotes mRNA translation. The observed upregulation of mRNA translation appears to be a transient process associated with formation of active eIF4F complexes because the overall transcript levels for the relevant components vary slightly between cells cultured normally versus cells cultured under SUMO-2 overexpression conditions.

During mRNA translation, the rate-limiting step of translation initiation is mediated by the cap-binding protein eIF4E [23]. Post-translational modification and phosphorylation events are essential for the translation promoting function of eIF4E. These include phosphorylation of eIF4E [24]–[27] and its inhibitory binding protein 4E-BP1 (PHAS-I) [28]–[32]. When phosphorylated, 4E-BP1 releases eIF4E to form the eIF4F complex and promote translation [30], [32]. In addition to phosphorylation, sumoylaiton also plays an important role in this process. Xu et al. demonstrated that eIF4E modified by SUMO-1 can promote mRNA translation [11]. Moreover, the E3 ligase HDAC2 was shown to promote eIF4E sumoylation [33]. Here, our study showed that overexpression of SUMO-2 resulted in a marked increase in translation and a selective enhancement of key regulators of cell cycle progression, including c-myc and cyclin D1.

As a reversible post-translational modification, the SUMO family has gained prominence as a major regulatory component that impacts numerous aspects of cell growth and differentiation. Many experiments indicate that SUMO modification is important for passage through the cell cycle [34]–[37]. Sumoylation is also implicated in other forms of cellular growth control, such as senescence and apoptosis [38]–[40]. Moreover, the results of the studies described earlier indicate that sumoylation is not only an important regulator of the normal function of many vital cellular proteins but also a contributor in the pathogenesis of some human diseases. For instance, several lines of evidence point to a role of the SUMO modification pathway in tumorigenesis [41], [42]. Ubc9 overexpression can increase cancer cell growth [43], [44]. The SUMO E3 protein PIAS3 is upregulated in several different cancer types [45], and elevated levels of the SUMO E1 enzyme are associated with more severe hepatocellular carcinomas [46]. Additionally, deletion of the protease genes, like Ulp1in yeast, stops cell cycle progression and further highlights the essential and critical role of sumolation in the cell's life cycle [47]. Overexpression of SUMO-2 and the Uba2 E1 subunit has been correlated with poor survival of hepatocellular carcinoma patients [46]. In our study, the cell cycle and apoptosis assays indicate that overexpression of SUMO-2 promotes cell cycling and inhibits apoptosis. On the other hand, SUMO-2 depletion inhibits the cell cycle and promotes apoptosis, which is consistent with previous studies. We suspect that the observations from our experiments may be derived from the following two aspects. On one hand, many cell cycle- and apoptosis-related proteins and kinases are thought to be the targets of SUMO-2 [48], so it is clear that SUMO-2 modification plays an important regulatory role in these cell processes. On the other hand, many cell cycle- and apoptosis-related proteins are also cap-dependent proteins, which suggests that faster or slower cell cycle and apoptosis may result from higher or lower expression levels of these proteins. We hypothesize that both of them contribute to this phenomena. Thus, our results further explain the relationship between sumoylation and human diseases.

Over the past ten years, SUMOs have been established as essential regulators of many cellular functions. Aberrant SUMO regulation is a likely cause of a variety of human diseases. While new SUMO targets are rapidly identified, many fundamental questions remain unanswered. Our studies show that, like other components in sumoylation circulation, SUMO-2 can also promote mRNA translation by regulating the formation of the eIF4F complex. We believe that our findings could be relevant to how SUMO-2 participates in physiological and pathological processes in cells.
