# Correction: SUMO-2 Promotes mRNA Translation by Enhancing Interaction between eIF4E and eIF4G

**DOI:** 10.1371/journal.pone.0108832

**Published:** 2014-09-17

**Authors:** The PLOS ONE Staff

The 11^th^,12^th^, and 13^th^ authors are listed out of order. The correct order is: Li-zhao Chen ^1,2,#^, Xiang-yun Li ^1,3,#^, Hong Huang ^1^, Wei Xing ^1^, Wei Guo ^1^, Jing He ^3^, Zhi-ya Sun ^3^, An-xiong Luo ^3^, Hua-ping Liang ^1^, Jing Hu ^5^, Zheng-guo Wang ^4,*^, Yun-sheng Xu ^6,*^, Xiang Xu ^1,3,*^

^1^First department, State Key Laboratory of Trauma, Burn and Combined Injury, Research Institute of Surgery and Daping Hospital, Third Military Medical University, Chongqing, China

^2^Department of Neurosurgery, Research Institute of Surgery and Daping Hospital, Third Military Medical University, Chongqing, China

^3^Cell-based Biotherapy Center, Research Institute of Surgery and Daping Hospital, Third Military Medical University, Chongqing, China

^4^Fourth department, State Key Laboratory of Trauma, Burn and Combined Injury, Research Institute of Surgery and Daping Hospital, Third Military Medical University, Chongqing, China

^5^Department of Pharmacology and Chemical Biology, University of Pittsburgh Cancer Institute, University of Pittsburgh School of Medicine, Pittsburgh, Pennsylvania, USA.

^6^Department of Dermatology, First Affiliated Hospital of Wenzhou Medical College, Wenzhou Zhejiang, China

#These authors contributed equally to this work.

*These authors are co-corresponding authors
